# Using economic modelling to contribute to the prioritisation and design and clinical trials: ready for prime time

**DOI:** 10.1186/1745-6215-12-S1-A40

**Published:** 2011-12-13

**Authors:** Mark Sculpher

**Affiliations:** 1 Centre for Health Economics, University of York, York, YO10 5DD, UK

## 

It is now generally recognised that health systems internationally have resource constraints and need to set priorities in selecting the interventions they fund. Hence considerations of value for money are central in health policy worldwide. Cost-effectiveness analysis is a formal assessment of value, and is central to how many health care systems make resource allocation decisions. The purpose of clinical trials is to generate evidence to support various types of decision making, and an increasing number of trials are designed primarily to inform health system decisions (e.g. the NIHR Health Technology Assessment Programme in the UK).

Consequently, the process of designing a clinical trial and determining whether it is a priority for research funding needs an explicit consideration of whether it can contribute to better decisions in the future. This need to tie trials to ultimate decisions about cost effectiveness is at odds with the role that economic analysis has generally assumed in clinical trials over the last 20 years - i.e. trials being designed largely to address clinical questions but offering some add-on economic data to facilitate some form of cost effectiveness analysis when the trial reports.

Analytical methods exist which assess the value of trials in terms of the likelihood of their improving resource allocation decisions. Based on Bayesian decision theory, these methods quantify the uncertainty relating to the most cost effective approach to managing a specific patient group based on existing evidence, couple this with factors such as the size of the patient population to estimate the expected value of perfect information which can be used to begin prioritising trials. Extensions to these methods consider the appropriateness of specific trial designs by assessing their marginal costs and benefits in terms of reduction in the cost of decision uncertainly. These methods have been used within the HTA Programme and there are examples of impact in trial funding decisions. They are, however, perceived as being complex and requiring significant analysis. Although this perception can be challenged, there is undoubtedly a need for methods that can be applied routinely to assess the potential trial value. These might include the need for all trial proposals to include modelling to establish a plausible effect size which is sufficient to demonstrate an intervention's cost-effectiveness in the context of all existing evidence. This modelling would also be used to justify design features such as the choice of comparators, endpoints and follow-up periods.

